# Subacute edema progression after acute ischemic stroke: impact of intravenous alteplase administration and reperfusion degree

**DOI:** 10.3389/fneur.2025.1698480

**Published:** 2025-11-25

**Authors:** Wiktor Olszewski, Fabiano Cavalcante, Laura van Poppel, Ludo Beenen, Bart J. Emmer, Ido van den Wijngaard, Robin Lemmens, Yvo Roos, Henk Marquering, Praneeta Konduri, Charles Majoie

**Affiliations:** 1 Department of Biomedical Engineering and Physics, Amsterdam UMC - Location University of Amsterdam, Amsterdam, Netherlands; 2 Department of Radiology and Nuclear Medicine, Amsterdam UMC - Location University of Amsterdam, Amsterdam, Netherlands; 3 Department of Neurology and Radiology, Haaglanden Medical Center, The Hague, Netherlands; 4 Department of Neurology, Leiden University Medical Center, Leiden, Netherlands; 5 KU Leuven, Department of Neurosciences, Experimental Neurology, Leuven, Belgium; 6 University Hospitals Leuven, Department of Neurology, Leuven, Belgium; 7 Department of Neurology, Amsterdam UMC - Location University of Amsterdam, Amsterdam, Netherlands; 8 Department of Neurology and Neurological Sciences, Stanford University, Palo Alto, CA, United States

**Keywords:** cerebral edema, progression, ischemic lesion, net water uptake, subacute, acute ischemic stroke

## Abstract

**Introduction:**

Alteplase is known to increase the risk of blood-brain barrier integrity disruption, potentiating hemorrhage and edema. Evolving edema reduces chances of good functional outcomes. There is a paucity of studies that investigate the role of alteplase administration in subacute edema progression. Here we aim to associate alteplase administration in combination with the degree of reperfusion on edema, measured by net water uptake.

**Methods:**

We included 115 patients from the MRCLEAN NO-IV trial with baseline, 24-h and 1-week follow-up non-contrast CT scans. The cohort consisted of patients who received intravenous thrombolysis (IVT)+ endovascular treatment (EVT) vs. EVT alone. Net water uptake (NWU) was calculated as a ratio of mean lesion density compared to its homologous, contralateral region-of-interest. Unadjusted linear regression analysis was performed to assess the association between NWU progression and alteplase administration, successful reperfusion [expanded Thrombolysis in Cerebral Infarction (eTICI)2B/3], and excellent reperfusion (eTICI2C/3). Adjusted regression analysis was performed to correct for potential confounders.

**Results:**

IVT administration was not statistically significantly associated with NWU progression. Regardless of treatment arm, there was substantial increase in NWU during the first 24 h and 1 week post-stroke. In adjusted analysis, successful reperfusion was significantly associated with reduced NWU progression at 24 h (β = −4.6; 95% CI: −8.4, −0.80) and 1 week (β = −6.5; 95% CI: −11, −2.3).

**Conclusion:**

Alteplase administration prior to EVT did not impact the subacute edema progression in our cohort, whereas successful reperfusion was strongly associated with reduced edema progression, particularly at later timepoints. These results suggest that alteplase administration according to current guidelines is unlikely to contribute to accelerated edema progression and emphasize that achieving high-grade reperfusion is crucial for reducing secondary injury.

## Introduction

The added value of intravenous thrombolysis (IVT) with alteplase as an addition to endovascular treatment (EVT) has been questioned. Yet, results from recent studies indicate that administering IVT before EVT results in similar outcomes to EVT alone (1), but the evidence is not definitive. Pooled data analyses suggest that the benefit of IVT may be time-dependent, with greater effect when administered shortly after stroke onset (2). Kaesmacher et al. found that the benefit of IVT in addition to EVT decreased with increasing onset-to-treatment time and was statistically significant only if administered within 2 h and 20 min after onset. Nevertheless, uncertainty remains about the contribution of adding IVT on the increase of cerebral edema (3).

Cerebral edema may evolve even after successful treatment (4, 5), at least up to 1 week after stroke onset (4), and can range from mild swelling to severe, life-threatening forms. Known risk factors for the development of severe or malignant edema include younger age, higher National Institutes of Health Stroke Scale (NIHSS) scores, and larger areas of parenchymal hypoattenuation on computed tomography (6). Malignant edema has also historically been suggested to occur more frequently in patients with cardioembolic stroke (7), although the evidence remains limited and inconclusive. Late lesion progression is associated with less favorable functional outcome (8). Post-treatment lesion progression could be attributed to secondary injury (such as cerebral edema) and/or the poor rates of microvascular reperfusion referred to as no-reflow phenomenon (9). However, Konduri et al. (5) reported that successful treatment is associated with reduced edema progression. Within the lesion, tissue density is altered due to water migration resulting from disruption of the osmotic gradient. Minnerup et al. developed a method of quantifying the change in the water content in the tissue, known as net water uptake (NWU) (10).

Alteplase, or rtPA, is a serine protease that triggers degradation of plasminogen to proteinase plasmin, which may dissolve the occluding thrombus and restore perfusion. While the clinical utility of this treatment is well-established, it is known from animal models that alteplase can affect integrity of the blood-brain barrier and induce hemorrhages (11). Beyond the risk of hemorrhagic complications, alteplase has been suggested to contribute to the development and progression of cerebral edema (12). Disruption of the blood-brain barrier leads to increased vascular permeability, extravasation of plasma proteins, and promoting vasogenic edema. Cerebral edema after stroke has been repeatedly associated with worse clinical outcomes (5, 13), and its progression could reflect ongoing injury, despite successful reperfusion. However, the effect of alteplase on cerebral edema progression in the subacute period remains unclear.

Understanding whether alteplase plays a role in edema progression may have implications for clinical management. While alteplase could potentially exacerbate edema formation and progression by disturbing the blood-brain barrier, it may also mitigate edema by promoting lysis of microthrombi and improving microvascular reperfusion (14). If alteplase exacerbates edema formation and progression, it could support the use of adjunctive therapies, such as osmotic agents, to mitigate secondary injury in selected patients. Although no neuroprotective therapies have yet proven effective, research in this area remains active, exploring a wide range of strategies (15).

Here, we investigated the effects of IVT with alteplase on edema progression during the subacute period (24 h and 1 week after stroke onset) using NWU measurements. Additionally, we performed a secondary analysis to examine whether edema progression varies with recanalization success, which is assessed with the eTICI score.

## Methods

### Study population

MRCLEAN NO-IV is a multicenter, randomized clinical trial that evaluated whether EVT alone is superior to combined IVT and EVT in acute ischemic stroke (AIS) patients with proximal intracranial LVO admitted directly to an EVT capable center (16, 17). Patients included in the study were over 18 years old, met criteria for receiving IVT with alteplase, and presented at a medical center that offered both treatments. Patients were randomized to receive either EVT alone or EVT preceded by IVT. We included patients with available NCCT at admission and 24 h and 1 week after treatment. Patients with NCCTs with excessive contrast extravasation, poor quality, movement artefacts, or other technical errors were excluded. Patients who underwent decompressive craniectomy were also excluded. Details can be found in Supplementary material (Section 2). Given that a substantial number of patients from the original trial were excluded due to imaging availability, we assessed the potential for selection bias by comparing baseline characteristics between included and excluded patients. This comparison is presented in the Supplementary material (Section 3).

Approval for the MRCLEAN NO-IV trial was obtained from the central medical ethics committee and the research boards of all participating centers. Written informed consent was provided by all patients or their legal representatives. Data supporting the findings of this study are available from the corresponding author and CONSTRAST consortium (https://www.contrast-consortium.nl/data-requests-consortium-members-and-trial-collaborators/) upon reasonable request.

### Imaging assessment

A deep learning-based model based on a 3D nnUNet architecture was used to segment ischemic regions on baseline NCCT, with CTA images incorporated as additional input to inform the segmentation. The model was trained on annotated imaging data from the MRCLEAN Registry, MRCLEAN-MED, and MRCLEAN-LATE datasets, with MRCLEAN NO-IV cases excluded from training and validation to prevent bias (18).

The methodology to segment infarct lesions in follow-up NCCT scans (24-h and 1-week) has been described earlier (19). In summary, ischemic and hemorrhagic regions were segmented using a deep learning-based software developed by Nicolab (20). The segmented lesions were visually assessed and, if needed, manually corrected using ITK-SNAP software (21). Assessors were blinded for clinical data except for symptomatic side. The lesion delineations encompassed hypodensities that extended into the contralateral hemisphere or led to the deformation/compression of ventricles or sulci, as well as hyperdensities within/adjacent to the hypodense brain regions suspected as hemorrhage or calcifications. All segmentations were manually corrected by a trained neurologist (>5 years of experience) after consulting with an experienced neuro-radiologist (>15 years of experience) when necessary. Hemorrhages were outlined and excluded from the total lesion segmentation.

Non-hemorrhagic lesion volume (infarct and edema) where obtained by subtracting hemorrhagic regions from the total lesion segmentation. All other radiological and treatment parameters were assessed by the central blinded core-lab (16).

### Net water uptake

**Figure 1:**
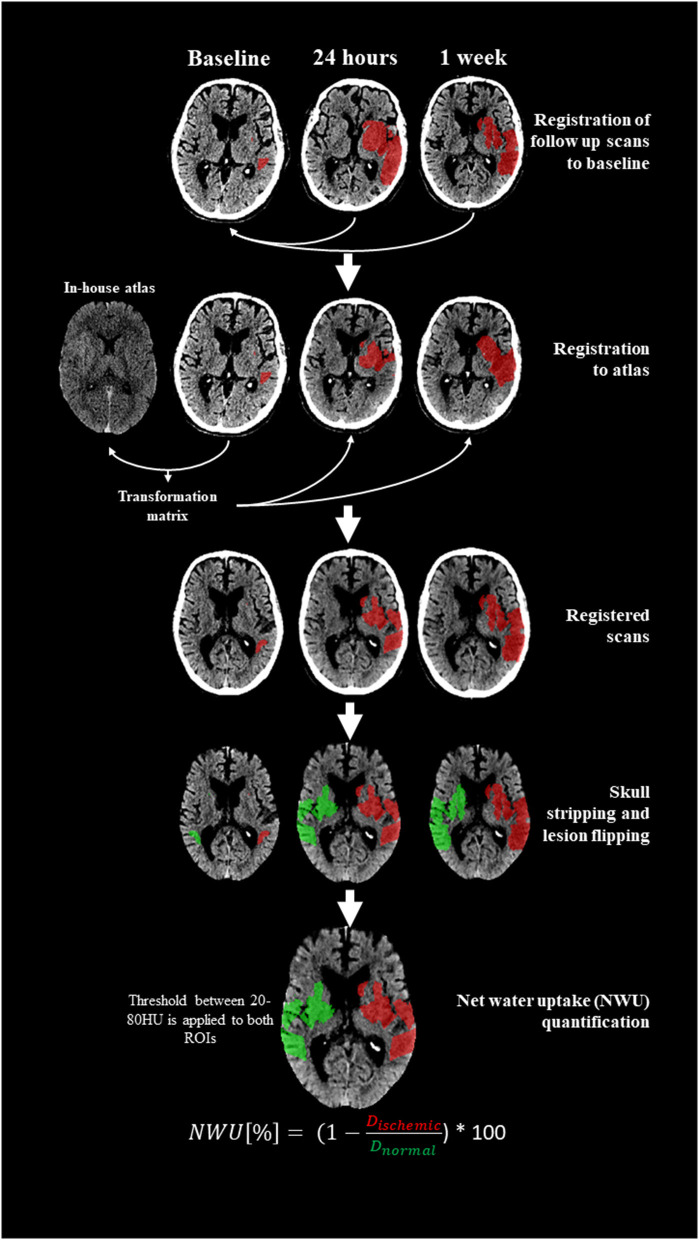
NWU processing pipeline. Follow-up scans were first registered to baseline. Baseline scans were subsequently aligned to an in-house atlas. The latter transformation matrices were applied to follow-up scans. Ischemic lesion segmentations were mirrored onto the contralateral hemisphere and a threshold of 20–80 HU was applied to define a reference region, and NWU was calculated as the ratio of mean lesion density to its contralateral counterpart.

NWU is calculated as the ratio of the mean density of the infarct lesion to that of its contralateral region-of-interest, considering only voxels with a density between 20 and 80 Hounsfield Units (HU) (22). To perform this calculation, the segmented ischemic lesion is mirrored onto the corresponding region in the contralateral (non-affected) hemisphere (Figure 1).

To ensure that all images were aligned in a standardized space and allow for reliable mirroring of ischemic lesion segmentations, firstly, the follow-up scans (both 24-h and 1-week) were registered to their corresponding baseline scan, using affine transformation. Secondly, the baseline scans were registered to an in-house atlas to establish a common reference space. The transformation matrices acquired in this step were then applied to the previously aligned follow-up scans, ensuring all images for the individual subject occupied the same space.

Following registration and segmentations mirroring, the NWU is calculated as the ratio of the mean density of the ischemic lesion (D_ischemic_) in HU to the mean density of the contralateral, healthy tissue (D_pre − ischemic_) as outlined in the formula below:


$$\begin{array}{l} NWU\;\left[\%\right]=\left(1-\frac{D_{ischemic}\left[HU\right]}{D_{pre-ischemic}\left[HU\right]}\right)*100\% \end{array}$$

Higher NWU values indicate greater water uptake and more pronounced ischemic edema. Note that NWU is dimensionless and ranges between 0 and 100%.

### Statistical analysis

In our study, NWU progression is the parameter of interest. However, using the change in a parameter (such as NWU increase) as a dependent variable can lead to biased results (23). For this reason, we created a model to estimate the follow-up NWU (NWU_FU_) with baseline NWU (NWU_BL_) as an independent parameter.


$$\begin{array}{l} NWU_{FU}=B_{0}+B_{1}\cdot NWU_{BL}+B_{2}\cdot IVT \end{array}$$

Where B_i_ are the parameters describing the strength of relation of NWU_BL_ and IVT treatment (yes/no). This approach aimed to minimize bias related to baseline imbalance. Subsequently, we reformulated the model to express the progression ΔNWU, rather than absolute NWU value at follow-up.


$$\begin{array}{l} \Delta NWU=NWU_{FU}-NWU_{BL}\\ \Delta NWU=B_{0}+\left(B_{1}-1\right)\cdot NWU_{BL}+B_{2}\cdot IVT \end{array}$$

The influence of IVT administration on NWU at 24-h (NWU_24h_) and 1-week (NWU_1wk_) post-stroke using univariable and multivariable linear regression after adjusting for age, sex, baseline NIHSS, baseline Alberta Stroke Programme Early CT Score (ASPECTS), reperfusion status (eTICI2B/3 or eTICI2C/3), and onset-to-randomization time.

Given that the reperfusion degree may influence edema progression, we conducted a secondary analysis to examine this relationship using univariable and multivariable linear regression models. We stratified patients based on their achieved reperfusion status. First, we categorized successful reperfusion as eTICI ≥ 2B. Subsequently, to assess the impact of excellent reperfusion, we repeated the analysis using a stricter threshold, considering successful reperfusion as eTICI2C/3.

Dichotomous and categorical baseline characteristics are presented as proportions. Continuous variables are presented as median and interquartile ranges. Mann–Whitney *U*-test and Chi-Square/Fisher-tests were performed to compare continuous and binary/categorical variables. Missing data was imputed using Multivariate Imputation by Chained Equations (MICE), implementing 20 imputations. The details of the MICE imputation are provided in Section 1 of the Supplementary material. Significance was assessed at a threshold of *p* < 0.05 and results were reported as adjusted estimates with corresponding 95% confidence intervals. Statistical analyses were performed using R (4.3.3[2024-02-29]).

## Results

Of the 539 patients in the MRCLEAN NO-IV trial, 115 met the inclusion criteria (see Section 2 in Supplementary material). Median age was 71 (IQR: 59–76) years, 73 patients (63%) were male, and the median baseline NIHSS score was 16 (IQR: 11–19). Successful reperfusion (eTICI2B-3) was achieved in 96 patients (83%). Sixty-six patients (57%) achieved eTICI2C/3. Sixty-eight (59%) patients received IVT, and the median onset-to-randomization time was 92 (IQR: 70–140) minutes (Table 1).

**Table 1:** Baseline characteristics after multivariate imputation by chained equations (MICE).

**Variable**	**Population (*n* = 115)**	**EVT alone (*n* = 47)**	**IVT and EVT (*n* = 68)**	***p*-value**
Age (years)	71 (59–76)	73 (65–80)	67 (58–75)	0.04
Sex (male)	73 (63%)	30 (64%)	43 (63%)	1
History of ischemic stroke	13 (11%)	7 (15%)	6 (9%)	0.48
History of atrial fibrillation	18 (16%)	9 (19%)	9 (13%)	0.55
History of diabetes mellitus	21 (18%)	10 (21%)	11 (16%)	0.65
History of hypertension	50 (43%)	22 (47%)	28 (41%)	0.68
Pre-stroke mRS > 2	3 (3%)	2 (4%)	1 (1%)	0.74
Baseline glucose (mmol/L)	6.7 (5.9–8.1)	6.8 (5.8–7.8)	6.6 (5.9–8.8)	0.56
Baseline systolic blood pressure (mmHg)	150 (130–170)	160 (130–170)	150 (130–170)	0.97
Onset-to-randomization time (min)	92 (70–140)	98 (70–140)	90 (70–140)	0.90
Door-to-groin time (min)	69 (54–95)	65 (52–90)	70 (57–96)	0.48
Onset-to-groin time (min)	140 (110–190)	150 (110–180)	140 (110–200)	0.98
Onset-to-reperfusion time (min)	180 (160–230)	190 (160–250)	180 (160–220)	0.76
Right-sided stroke	57 (50%)	25 (53%)	32 (47%)	0.65
**Stroke subtype according to TOAST classification**				
Cardioembolic	32 (28%)	18 (38%)	14 (21%)	0.19
Large artery atherosclerosis	19 (17%)	8 (17%)	11 (16%)	
Other determined	1 (1%)	0 (0%)	1 (1%)	
Undetermined etiology	57 (50%)	18 (38%)	39 (57%)	
Undetermined etiology (more than one cause)	6 (5%)	3 (6%)	3 (4%)	
Baseline NIHSS	16 (11–19)	16 (11–19)	16 (9–19)	0.76
Baseline ASPECTS	9 (8–10)	9 (8–10)	9 (8–10)	0.92
Proximal occlusion (MCA)	95 (83%)	38 (81%)	57 (84%)	0.87
24-h mAOL score = 3	92 (80%)	36 (77%)	56 (82%)	0.60
**Baseline collateral score**				
Score 0 (absent collaterals)	6 (5%)	2 (4%)	4 (6%)	0.50
Score 1 (filling ≤ 50% occluded area)	31 (27%)	13 (28%)	18 (26%)	
Score 2 (>50%; < 100%)	51 (44%)	24 (51%)	27 (40%)	
Score 3 (100% occluded area)	27 (23%)	8 (17%)	19 (28%)	
eTICI2B-3	96 (83%)	42 (89%)	54 (79%)	0.25
eTICI2C-3	66 (57%)	28 (60%)	38 (56%)	0.84
90-day mRS ≤ 2	56 (49%)	21 (45%)	35 (51%)	0.60
**90-day mRS**				
0	3 (3%)	1 (2%)	2 (3%)	0.11
1	10 (9%)	5 (11%)	5 (7%)	
2	43 (37%)	15 (32%)	28 (41%)	
3	11 (10%)	7 (15%)	4 (6%)	
4	18 (16%)	3 (6%)	15 (22%)	
5	15 (13%)	7 (15%)	8 (12%)	
6	15 (13%)	9 (19%)	6 (9%)	
Baseline lesion volume (mL)	14 (3.7–34)	11 (2.5–26)	15 (5.5–37)	0.13
Baseline edema (mL)	0.5 (0.1–1.5)	0.5 (0.1–1.4)	0.6 (0.2–1.8)	0.21
Baseline NWU (%)	4.3 (2.1–6.8)	4 (1.8–6.7)	4.5 (2.4–6.7)	0.63
Hemorrhagic transformation	41 (36%)	17 (36%)	24 (35%)	1

Data displayed as median (interquartile ranges) or number (% of population). Mann-Whitney *U*-test and Chi-Square/Fisher tests were performed to compare continuous and binary/categorical variables between the sub-groups based on treatment allocation (EVT alone/EVT + VT).

Most baseline characteristics did not differ significantly between IVT + EVT vs. EVT alone patients. However, patients in the EVT+IVT group were younger than those in the EVT alone group (67 vs. 73 years; *p* = 0.04). There were no statistically significant differences in baseline NIHSS scores (median score of 16 in both groups; *p* = 0.76) or ASPECTS (median score of 9 in both groups; *p* = 0.92). Slightly more patients in the EVT alone group achieved successful reperfusion (89 vs. 79%; *p* = 0.25). There were no significant differences between groups in lesions characteristics: patients in the EVT alone group had smaller lesions [11 mL (IQR: 2.5–26) vs. 15 mL (IQR: 5.5–37); *p* = 0.13] and slightly lower NWU [4% (IQR: 1.8–6.7) vs. 4.5% (IQR: 2.4–6.7) in the EVT + IVT group; *p* = 0.67] at admission.

**Figure 2:**
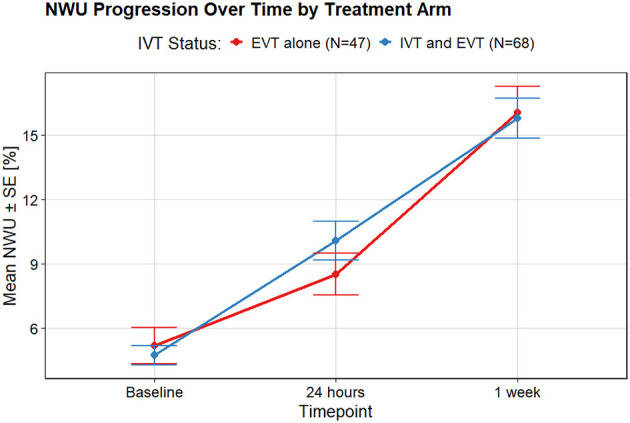
NWU progression over time for two treatment arms: EVT alone and IVT prior to EVT. In the first 24 h, edema extended more for patients who received IVT. This difference reduces within 1 week.

In both groups, median NWU increased substantially from baseline (EVT + IVT: 4.5%; EVT alone: 4%) to 24 h (EVT + IVT: 9.8%; EVT alone: 8.3%) and further to 1 week (EVT + IVT: 15%; EVT alone: 15%). NWU progression in the first 24 h was larger in the EVT + IVT group compared to EVT alone, but the difference was not statistically significant (Figure 2). For both the NWU progression during the first 24 h and 1 week NWU progression, the contribution of IVT administration was not statistically significant. In the multivariable models, IVT administration was also not significantly associated with NWU progression at either timepoint.

**Figure 3:**
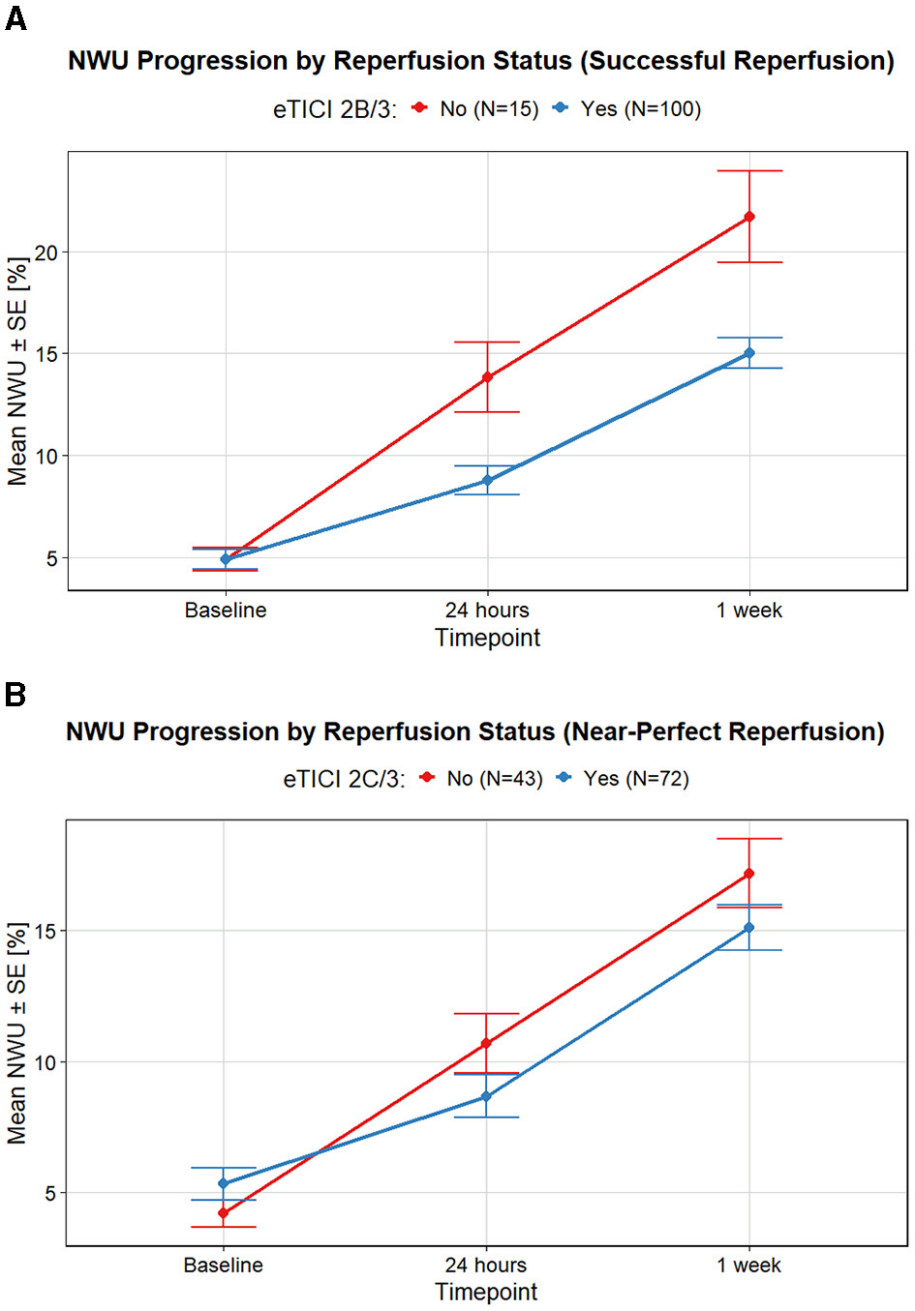
Comparison of NWU progression stratified by reperfusion status. **(A)** NWU progression for successful reperfusion (eTICI ≥ 2B) vs. unsuccessful reperfusion, showing significantly less edema progression in the successfully reperfused group. **(B)** NWU progression in patients with near-complete reperfusion (eTICI ≥ 2C) compared to those with lower reperfusion scores.

Successful reperfusion (eTICI2B/3) was statistically significantly associated with reduced NWU progression at both timepoints. However, this association between reperfusion status and NWU progression was no longer statically significant at 1 week follow-up if the threshold was set at eTICI2C/3 (Table 2; Figure 3).

**Table 2:** Univariate and multivariable linear regression analysis for predicting NWU progression at 24-h and 1-week follow-up.

**Predictor variable**	**Δ NWU_24h_**	**Δ NWU_1week_**
	β **(95%CI)**, ***p*****-value**	β **(95%CI)**, ***p*****-value**
**Association of IVT administration with** Δ**NWU**		
IVT administration	1.5 (−1.3, 4.2), *p* = 0.31	−0.4 (−3.5, 2.8), *p* = 0.83
**Association of eTICI2B/3 with** Δ**NWU**		
eTICI2B/3	–**4.7 (– 8.3, –1.1)**, ***p*** **=** **0.01**	–**6.4 (–10.5, –2.4)**, ***p*** **=** **0.002**
**Association of eTICI2C/3 with** Δ**NWU**		
eTICI2C/3	–**2.9 (–5.6, –0.1)**, ***p*** **=** **0.04**	−2.8 (−5.9, 0.4), *p* = 0.09
**Multivariable models**		
IVT administration	1.3 (−1.4, 4.1), *p* = 0.34	−0.7 (−3.9, 2.5), *p* = 0.66
Age	0.01 (−0.1, 0.1), *p* = 0.88	0.02 (−0.1, 0.2), *p* = 0.77
Sex (male)	1.9 (−1.0, 4.8), *p* = 0.19	0.6 (−2.7, 3.9), *p* = 0.73
Baseline NIHSS	0.1 (−0.1, 0.4), *p* = 0.24	0.1 (−0.2, 0.4), p = 0.48
Baseline ASPECTS	0.2 (−0.7, 1.1), *p* = 0.68	−0.1 (−1.1, 1.0), *p* = 0.90
eTICI2B/3	–**4.6 (–8.3, –1.0)**, ***p*** **=** **0.01**	–**6.6 (–10.8, –2.4), p** **=** **0.003**
Onset-to-randomization time	**0.02 (0.01, 0.04)**, ***p*** **=** **0.01**	0.02 (−0.00, 0.04), *p* = 0.06

Data displayed as median (interquartile ranges) or number (% of population). Mann-Whitney *U*-test and Chi-Square/Fisher tests were performed to compare continuous and binary/categorical variables between the sub-groups based on treatment allocation (EVT alone/EVT + VT). Values in bold denote statistically significant associations (*p* < 0.05).

In multivariable models, successful reperfusion (eTICI2B/3) was significantly associated with decreased NWU at both timepoints (Table 2). For the definition of reperfusion status as eTICI2C/3 (Table 3), this association was no longer significant for either timepoint.

**Table 3:** Multivariable regression analysis stratified by eTICI2C/3.

**Predictor variable**	**Δ NWU_24h_**	**Δ NWU_1week_**
	β **(95%CI)**, ***p*****-value**	β **(95%CI)**, ***p*****-value**
**Multivariable models**		
IVT administration	1.7 (−1.1, 4.4), *p* = 0.24	−0.2 (−3.5, 3.1), *p* = 0.90
Age	0.004 (−0.1, 0.1), *p* = 0.95	0.02 (−0.1, 0.2), *p* = 0.82
Sex (male)	1.4 (−1.5, 4.3), *p* = 0.34	−0.1 (−3.5, 3.3), *p* = 0.95
Baseline NIHSS	0.1 (−0.1, 0.4), *p* = 0.34	0.1 (−0.2, 0.3), *p* = 0.66
Baseline ASPECTS	0.1 (−0.8, 1.0), *p* = 0.84	−0.1 (−1.2, 0.9), *p* = 0.80
eTICI2C/3	−2.5 (−5.3, 0.3), *p* = 0.08	−2.6 (−5.9, 0.7), *p* = 0.12
Onset-to-randomization time	**0.02 (0.01, 0.04)**, ***p*** **=** **0.01**	0.02 (−0.001, 0.04), *p* = 0.06

## Discussion

In this study, we showed that in EVT-treated patients, edema strongly increases at both 24-h and 1-week follow-up post-stroke, irrespective of alteplase administration. We also showed that successful reperfusion, defined as eTICI2B/3, is associated with reduced edema progression, also after adjusting for confounders. While in the univariate analysis (near-)perfect (eTICI2C/3) reperfusion was associated with reduced edema progression at 24-h follow-up, in the adjusted analysis we found no significant association at either imaging time.

Adverse effects of IVT have been described previously (3). Yet, there is little knowledge on the effect of IVT on subacute edema progression, quantified as NWU, in the subacute time window from stroke onset. Unlike methods dependent on global anatomical changes, NWU is well-suited for assessing edema extent even in small infarcts. Even though no statistically significant association between IVT and NWU progression had been observed, patients who received IVT alongside EVT, on average, showed greater edema progression within the first 24 h, though not significant. In their study, Frisullo et al. defined edema progression as the increase in total lesion volume over time. While this volumetric approach provides an estimate of tissue expansion, it may be influenced by factors such as infarct growth, making it challenging to isolate edema-specific changes. In contrast, our study utilized a densitometric approach, giving a direct indicator of tissue water content. This method allows for a more precise edema progression quantification by capturing changes in tissue composition.

In our cohort, we did not observe a significant difference in cerebral edema progression between patients who received alteplase and those who did not, suggesting that alteplase administration does not exacerbate or mitigate edema progression after AIS. While alteplase is well-established as an effective treatment, it is also known to carry risks such as intracerebral hemorrhage or angioedema (3), which must be weighed against its benefits. Particularly for patients who are eligible for EVT, evidence from randomized controlled trials regarding the benefit of administering alteplase prior to EVT remains inconclusive (16, 24–28), suggesting that in this population the potential risks may outweigh the limited added value. However, results of a meta-analysis by Kaesmacher et al. (2) suggested that the benefit of alteplase administration might be time dependent. In their study, the addition of IVT prior to EVT within 2 h and 20 min from stroke onset resulted in better functional outcomes. In light of these results, consideration should be given to the time from onset when planning treatment. For patients presenting later within the recommended time window, the risk-to-benefit ratio may be less favorable as the therapeutic effect of IVT prior to EVT decreases the longer the delay from onset.

Late lesion progression has been shown to be most extensive in patients who did not achieve full reperfusion (29), indicating that insufficient oxygen and nutrients supply drives a cascade of pathological biophysical and mechanistic pathways resulting in an increased water uptake in the ischemic tissue. In situations where distal emboli limit microvascular reperfusion, strategies such as local administration of alteplase or tenecteplase after EVT (PEARL, ANGEL-TNK) have been explored to improve microvascular flow and potentially reduce infarct and edema progression with promising but not yet definitive results (30, 31). Lesion progression could also be caused by an abnormal osmotic gradient across intra- and extra-cellular spaces leading to cytotoxicity. Moreover, abnormal osmotic gradients and disruptions to the blood-brain barrier can induce vasogenic edema (32). Our cohort consisted of patients that received treatment within 4.5 h from onset, further emphasizing that that early reperfusion prevents tissue damage and subsequently limits edema development. Conversely, in more severe cases with greater ischemic injury, reperfusion injury may be more common, potentially exacerbating edema formation despite vessel recanalization (5). Discussions on the relationship between reperfusion and edema frequently center around the concept of reperfusion injury (33). Reperfusion injury refers to tissue damage that occurs when blood flow is restored to previously ischemic brain regions, triggering pathological processes such as apoptosis, inflammation, oxidative stress, and blood-brain barrier disruption, which can promote edema development (34). It is important to note that 83% of patients included in this study achieved successful reperfusion, resulting in a substantial imbalance within both treatment groups.

The association between successful reperfusion and reduced cerebral edema formation has been previously described by Brooks et al. (35); however, their findings were based on retrospective observational data and were limited to a 24-h time window. In contrast, our study assessed edema progression up to 1-week post-stroke. We also performed a subanalysis on the effect of near-complete reperfusion (eTICI2C/3) on edema progression. In this secondary analysis, the association between reperfusion status and NWU progression was only significant at 24 h follow-up, and no longer significant at either timepoint after adjusting for confounders. Knowing that higher eTICI scores are associated with better functional outcomes, it is a somewhat unexpected observation. One possible explanation may lie in the skewed distribution of reperfusion grades in our cohort, with 83% achieving eTICI ≥ 2B and 57% achieving ≥ 2C, which could limit statistical power of the analysis and reduce observed contrasts. Another possible explanation is that partial reperfusion preserves vessel wall integrity to a degree sufficient to limit edema progression. Further improvement from partial to near-complete reperfusion may contribute little additional effect on edema, although higher eTICI scores are still associated with better functional outcomes (36). Moreover, procedural factors such as the number of passes, thrombus location, or device choice could play a critical role in infarct progression and edema formation process. Since our analysis did not account for these intervention-related variables, the interpretability of these findings remains limited. Nevertheless, it is now known that achieving higher reperfusion status is positively associated with functional outcomes, and thus, should be sought after (36).

Our study has a few limitations. Firstly, the patient cohort included in this study was relatively small, which may have resulted in low statistical power. As a result, the potentially meaningful associations between IVT administration and edema progression could remain undetected. Limited power also reduces the precision of effect estimates, making it more difficult to draw definitive conclusions. Future pooled analyses including data from other randomized clinical trials investigating the effect of IVT in patients that can directly undergo EVT (IRIS collaboration) could provide more precise answers (1). Importantly, our analyses focus on imaging markers of edema progression, which primarily reflect biological processes. We intentionally did not address the relation of these imaging markers with functional outcome in the current study. Therefore, our findings should be interpreted in the context of edema biology rather than clinical recovery. Nonetheless, the prognostic value of NWU change remains an important topic of investigation. A subsequent study from our group, specifically modeling the association between NWU change and 90-day functional outcome is currently being finalized. Secondly, this study did not account for thrombectomy-related variables, such as the number of passes or device selection. These factors could influence endothelial damage and subsequent edema formation but were not included in our analysis. As a result, the impact of intervention-related variables on edema progression remains uncertain, and further research incorporating these aspects may provide a more comprehensive understanding of the relationship between reperfusion and edema. Future studies should also evaluate edema progression in patients of advanced age (≥85 years), as demographics, risk factors, and outcomes have been shown to differ in this age group (37). Thirdly, assessment of ischemic lesions on NCCT is not straight-forward, especially at baseline and 24-h follow-up, since the hypo-dense areas might not be clearly defined in this early time window. Moreover, in contrast to follow-up scans, lesion delineations at baseline were segmented using an automated method, and the accuracy of these segmentation was not manually assessed by a clinician. This could introduce segmentation errors, particularly given that infarcts on baseline NCCT are often subtle and challenging to delineate due to low contrast and indistinct lesion boundaries. Future studies should incorporate expert validation of automated segmentations to enhance accuracy and reliability. Lastly, our approach to quantifying NWU differs slightly from previous studies. In the original method, Minnerup et al. excluded all patients with hemorrhagic transformation (10). In contrast, we included patients with identified hemorrhagic transformation but removed hemorrhagic regions from the lesion segmentations. While this approach prevents inclusion of non-edematous tissue, it may lead to an NWU underestimation, as hemorrhages often occur in areas of severe blood-brain barrier disruption where water accumulation is also expected (38). Additionally, in cases of large hematomas (such as parenchymal hematoma type 2), surrounding edema may be compressed by the hemorrhage, reducing the apparent hypodense volume on CT and further contributing to a potential underestimation of NWU. However, including hemorrhagic regions in NWU calculations is not feasible, as the presence of blood alters tissue density in a way that does not accurately reflect water content, potentially confounding the measurement.

## Conclusion

In this study, a substantial increase in subacute cerebral edema was observed in all patients, regardless of treatment assignment. Alteplase administration prior to EVT did not influence this progression, whereas the degree of reperfusion remained an important determinant of edema dynamics. This suggests that the use of alteplase in accordance with current guidelines is unlikely to exacerbate cerebral edema and should not be withheld on this basis.

## Data Availability

The data analyzed in this study can be made available upon reasonable request to the CONSTRAST consortium. Requests to access these datasets should be directed to https://www.contrast-consortium.nl/data-requests-consortium-members-andtrial-collaborators/.
