# Supplementary material for: Subacute edema progression after acute ischemic stroke: impact of intravenous alteplase administration and reperfusion degree
Source: Front Neurol. 2025 Nov 25;16:1698480. doi: 10.3389/fneur.2025.1698480 (PMC12685625; doi:10.3389/fneur.2025.1698480)
Supplement: Supplementary file 1 [file Table_1.docx]

# Supplementary material

## Section 1

Missing data were addressed using multiple imputation by chained equations (MICE). Imputation was performed using 20 datasets (m = 20) and 5 iterations per imputation (maxit = 5). All other settings were kept at default. Variables were imputed according to their type: predictive mean matching (PMM) for numerical variables, a proportional odds model (POLR) for ordinal variables, and logistic regression for categorical variables. As this analysis was conducted using data from a single trial, no additional pooling was required. All variables included in the multiple imputation model, with the number and percentage of missing values for each variable are presented in the table below. Based on data inspection, missing values were assumed to be missing at random.

| Variable | Missing values |
| --- | --- |
| Age [years] | 0 (0%) |
| Sex [male] | 0 (0%) |
| Administration of IVT | 0 (0%) |
| History of ischemic stroke | 0 (0%) |
| History of atrial fibrillation | 0 (0%) |
| History of diabetes mellitus | 0 (0%) |
| History of hypertension | 0 (0%) |
| Pre-stroke mRS | 1 (1%) |
| Baseline lesion volume [mL] | 0 (0%) |
| Baseline edema [mL] | 0 (0%) |
| Baseline NWU [%] | 0 (0%) |
| Baseline glucose [mmol/L] | 3 (3%) |
| Baseline systolic blood pressure  [mmHg] | 0 (0%) |
| Onset-to-randomization time [min] | 0 (0%) |
| Door-to-groin time [min] | 4 (3%) |
| Onset-to-groin time [min] | 4 (3%) |
| Onset-to-reperfusion time [min] | 28 (24%) |
| Right-sided stroke | 0 (0%) |
| Baseline NIHSS | 0 (0%) |
| Baseline ASPECTS | 0 (0%) |
| Proximal occlusion (MCA) | 0 (0%) |
| 24-hour mAOL score | 13 (11%) |
| 24-hour NIHSS | 1 (1%) |
| 1-week NIHSS | 6 (5%) |
| Baseline collateral score | 1 (1%) |
| eTICI | 11 (10%) |
| 90-day mRS | 0 (0%) |
| Hemorrhagic transformation | 0 (0%) |
| 24-hour lesion volume [mL] | 0 (0%) |
| 24-hour edema [mL] | 0 (0%) |
| 24-hour NWU [%] | 0 (0%) |
| 24-hour hemorrhage volume [mL] | 0 (0%) |
| 1-week lesion volume [mL] | 0 (0%) |
| 1-week edema [mL] | 0 (0%) |
| 1-week NWU [%] | 0 (0%) |
| 1-week hemorrhage volume [mL] | 0 (0%) |

## Section 2

Flowchart describing the inclusion and exclusion criteria used in this study:


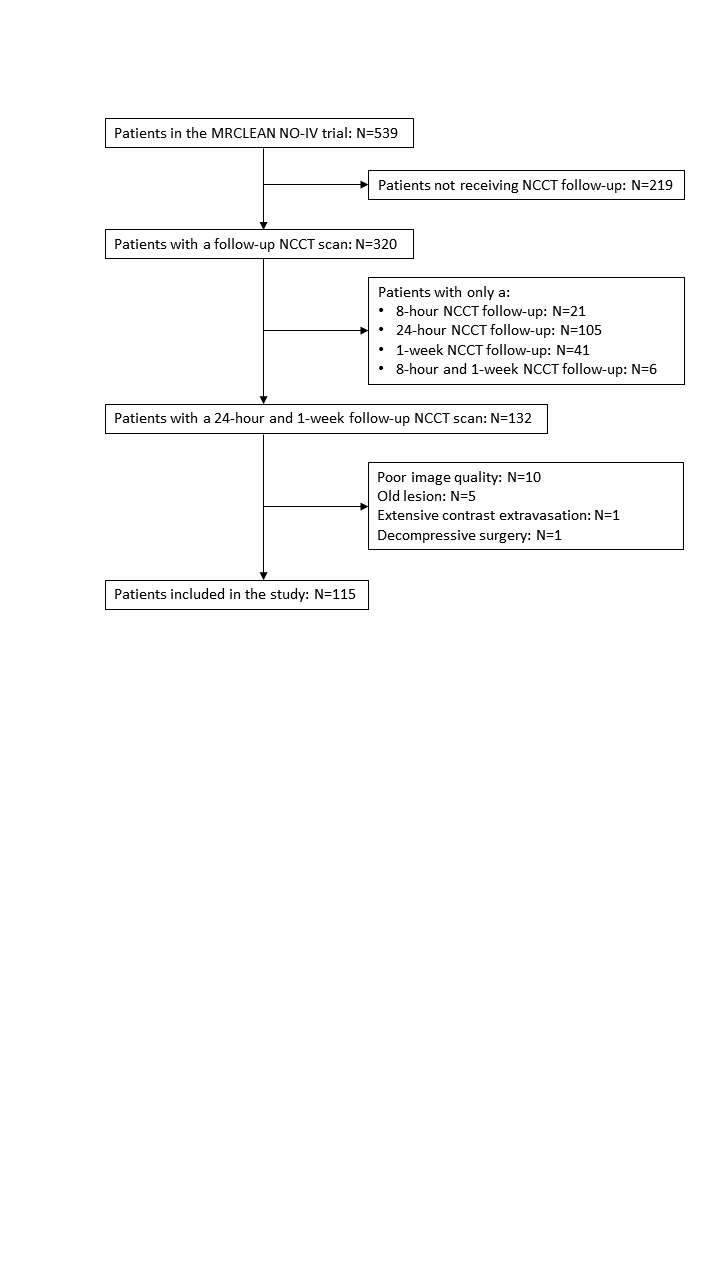


## Section 3

Comparison of baseline characteristics between patients included in this analysis and those excluded from the MRCLEAN NO-IV trial cohort. Data displayed as median (interquartile ranges) or number (% of population). Missing data is provided as number (% of population). Mann-Whitney U test and Chi-Square/Fisher tests were performed to compare continuous and binary/categorical variables between the sub-groups based on inclusion for this study.

| Variable | Population  (n=539) | Excluded  (n=424) | Included  (n=115) | p-value |
| --- | --- | --- | --- | --- |
| Age [years] | 71 (62-79) | 71 (62-80) | 71 (59-76) | 0.24 |
| Sex [male] | 303 (57%) | 230 (55%) | 73 (63%) | 0.11 |
| History of ischemic stroke | 91 (17%) | 78 (19%) | 13 (11%) | 0.48 |
| History of atrial fibrillation | 58 (11%) | 40 (10%) | 18 (16%) | 0.09 |
| History of diabetes mellitus | 90 (17%) | 69 (16%) | 21 (18%) | 0.74 |
| History of hypertension | 259 (48%) | 209 (50%) | 50 (43%) | 0.29 |
| Pre-stroke mRS>2 | 15 (3%) | 12 (3%) | 3 (3%) | 1.00 |
| Baseline glucose [mmol/L] | 6.7 (5.9-7.9) | 6.7 (5.9-7.8) | 6.6 (5.9-8.1) | 0.95 |
| Baseline systolic blood pressure  [mmHg] | 150 (130-170) | 160 (130-170) | 150 (130-170) | 0.52 |
| Onset-to-randomization time [min] | 93 (71-140) | 93 (71-150) | 92 (70-140) | 0.78 |
| Door-to-groin time [min] | **64 (50-78)** | **62 (50-75)** | **69 (53-86)** | **0.01*** |
| Onset-to-groin time [min] | 130 (100-180) | 130 (100-180) | 140 (110-190) | 0.09 |
| Onset-to-reperfusion time [min] | 180 (150-240) | 180 (140-230) | 190 (150-240) | 0.26 |
| Right-sided stroke | 248 (46%) | 191 (45%) | 57 (50%) | 0.49 |
| Stroke subtype according to TOAST classification | | | | |
| Cardioembolic | 136 (25%) | 104 (25%) | 32 (28%) | 0.55 |
| Large artery atherosclerosis | 76 (14%) | 57 (14%) | 19 (17%) |  |
| Other determined | 2 (0%) | 1 (0%) | 1 (1%) |  |
| Undetermined etiology | 298 (56%) | 241 (57%) | 57 (50%) |  |
| Undetermined etiology (more than one cause) | 24 (4%) | 18 (4%) | 6 (5%) |  |
| Baseline NIHSS | 16 (10-20) | 16 (10-20) | 16 (11-19) | 0.62 |
| Baseline ASPECTS | 9 (8-10) | 9 (8-10) | 9 (8-10) | 0.06 |
| Proximal occlusion (MCA) | 418 (78%) | 323 (77%) | 95 (83%) | 0.22 |
| 24-hour mAOL score=3 | 410 (76%) | 321 (76%) | 89 (77%) | 0.89 |
| Baseline collateral score | | | | |
| Score 0 (absent collaterals) | 32 (6%) | 26 (6%) | 6 (5%) | 0.92 |
| Score 1 (filling <= 50% occluded area) | 155 (29%) | 124 (29%) | 31 (27%) |  |
| Score 2 (>50%; <100%) | 230 (43%) | 179 (43%) | 51 (44%) |  |
| Score 3 (100% occluded area) | 119 (22%) | 92 (22%) | 27 (23%) |  |
| eTICI2B-3 | 428 (80%) | 331 (79%) | 97 (84%) | 0.22 |
| eTICI2C-3 | 308 (57%) | 242 (57%) | 66 (57%) | 1.00 |
| 90-day mRS≤2 | 268 (50%) | 212 (50%) | 56 (49%) | 0.83 |
| 90-day mRS | | | | |
| 0 | 26 (5%) | 23 (5%) | 3 (3%) | 0.27 |
| 1 | 57 (11%) | 47 (11%) | 10 (9%) |  |
| 2 | 185 (35%) | 142 (34%) | 43 (37%) |  |
| 3 | 52 (10%) | 41 (10%) | 11 (10%) |  |
| 4 | 63 (12%) | 45 (11%) | 18 (16%) |  |
| 5 | 55 (10%) | 40 (10%) | 15 (13%) |  |
| 6 | 98 (18%) | 83 (20%) | 15 (13%) |  |
| Hemorrhagic transformation | 167 (31%) | 126 (30%) | 41 (36%) | 0.29 |

## Section 4

The MR CLEAN-NO IV Investigators: Principal investigators: Yvo Roos (Amsterdam UMC, University of Amsterdam, Amsterdam, the Netherlands), Charles Majoie (Amsterdam UMC, University of Amsterdam, Amsterdam, the Netherlands), Study coordinators: Kilian Treurniet (Amsterdam UMC, University of Amsterdam, Amsterdam, the Netherlands), Jonathan Coutinho (Amsterdam UMC, University of Amsterdam, Amsterdam, the Netherlands), Bart Emmer (Amsterdam UMC, University of Amsterdam, Amsterdam, the Netherlands), Natalie LeCouffe (Amsterdam UMC, University of Amsterdam, Amsterdam, the Netherlands), Manon Kappelhof (Amsterdam UMC, University of Amsterdam, Amsterdam, the Netherlands), Leon Rinkel (Amsterdam UMC, University of Amsterdam, Amsterdam, the Netherlands), Agnetha Bruggeman (Amsterdam UMC, University of Amsterdam, Amsterdam, the Netherlands), Local principal investigators: Bob Roozenbeek (Erasmus MC University Medical Center, Rotterdam, the Netherlands), Adriaan van Es (Leiden Universitair Medisch Centrum, Leiden, the Netherlands), Inger de Ridder (Cardiovascular Research Institute Maastricht (CARIM), Maastricht University Medical Center, Maastricht, The Netherlands), Wim van Zwam (Cardiovascular Research Institute Maastricht (CARIM), Maastricht University Medical Center, Maastricht, The Netherlands), Bart van der Worp (University Medical Center Utrecht, Brain Center, Utrecht, the Netherlands), Rob Lo (University Medical Center Utrecht, Brain Center, Utrecht, the Netherlands), Koos Keizer (Catharina Hospital, Eindhoven, the Netherlands), Rob Gon (Catharina Hospital, Eindhoven, the Netherlands), Lonneke Yo (Catharina Hospital, Eindhoven, the Netherlands), Jelis Boiten (Haaglanden Medical Center, the Hague, the Netherlands), Ido van den Wijngaard (Haaglanden Medical Center, the Hague, the Netherlands), Geert Lycklama à Nijeholt (Haaglanden Medical Center, the Hague, the Netherlands), Jeannette Hofmeijer (Rijnstate Hospital, Arnhem, the Netherlands), Jasper Martens (Rijnstate Hospital, Arnhem, the Netherlands), Wouter Schonewille (St. Antonius Hospital, Nieuwegein, the Netherlands), Jan Albert Vos (St. Antonius Hospital, Nieuwegein, the Netherlands), Anil Tuladhar (Radboud University Medical Center, Nijmegen, the Netherlands), Floris Schreuder (Radboud University Medical Center, Nijmegen, the Netherlands), Jeroen Boogaarts (Radboud University Medical Center, Nijmegen, the Netherlands), Sjoerd Jenniskens (Radboud University Medical Center, Nijmegen, the Netherlands), Karlijn de Laat (HagaZiekenhuis, the Hague, the Netherlands), Lukas van Dijk (HagaZiekenhuis, the Hague, the Netherlands), Heleen den Hertog (Isala Klinieken, Zwolle, the Netherlands), Boudewijn van Hasselt (Isala Klinieken, Zwolle, the Netherlands), Paul Brouwers (Medisch Spectrum Twente, Enschede, the Netherlands), Emiel Sturm (Medisch Spectrum Twente, Enschede, the Netherlands), Tomas Bulut (Medisch Spectrum Twente, Enschede, the Netherlands), Michel Remmers (Amphia Hospital, Breda, the Netherlands), Anouk van Norden (Amphia Hospital, Breda, the Netherlands), Thijs de Jong (Amphia Hospital, Breda, the Netherlands), Anouk Rozeman (Albert Schweitzer Hospital, Dordrecht, the Netherlands), Otto Elgersma (Albert Schweitzer Hospital, Dordrecht, the Netherlands), Maarten Uyttenboogaart (University Medical Center Groningen, the Netherlands), Reinoud Bokkers (University Medical Center Groningen, the Netherlands), Julia van Tuijl (Elisabeth-TweeSteden Hospital, Tilburg, the Netherlands), Issam Boukrab (Elisabeth-TweeSteden Hospital, Tilburg, the Netherlands), Hans Kortman (Elisabeth-TweeSteden Hospital, Tilburg, the Netherlands), Vincent Costalat (Centre Hospitalier Universitaire de Montpellier, Montpellier, France), Caroline Arquizan (Centre Hospitalier Universitaire de Montpellier, Montpellier, France), Robin Lemmens (University Hospitals Leuven, Leuven, Belgium), Jelle Demeestere (University Hospitals Leuven, Leuven, Belgium), Philippe Desfontaines (Centre Hospitalier Chrétien, Liège, Belgium), Denis Brisbois (Centre Hospitalier Chrétien, Liège, Belgium), Frédéric Clarençon (Pitié-Salpêtrière hospital APHP-Sorbonne Université Paris France, Paris, France), Yves Samson (Pitié-Salpêtrière hospital APHP-Sorbonne Université Paris France, Paris, France), Local trial collaborators: Executive and writing committee: Diederik Dippel (Erasmus MC University Medical Center, Rotterdam, the Netherlands), Aad van der Lugt (Erasmus MC University Medical Center, Rotterdam, the Netherlands), Koos Keizer (Catharina Hospital, Eindhoven, the Netherlands), Jonathan Coutinho (Amsterdam UMC, University of Amsterdam, Amsterdam, the Netherlands), Bart Emmer (Amsterdam UMC, University of Amsterdam, Amsterdam, the Netherlands), Data Safety Monitoring Board: Martin Brown (National Hospital for Neurology and Neurosurgery, London, United Kingdom), Phil White (Institute of Neuroscience and Newcastle University Institute for Ageing, Newcastle University, Newcastle, UK), John Gregson (London School of Hygiene & Tropical Medicine, London, United Kingdom), Independent trial statistician: Daan Nieboer (Erasmus MC University Medical Center, Rotterdam, the Netherlands), Consortium coordinator: Rick van Nuland (Lygature, Utrecht, the Netherlands), Imaging assessment committee: Alida Postma (Cardiovascular Research Institute Maastricht (CARIM), Maastricht University Medical Center, Maastricht, The Netherlands), René van den Berg (Amsterdam UMC, University of Amsterdam, Amsterdam, the Netherlands), Ludo Beenen (Amsterdam UMC, University of Amsterdam, Amsterdam, the Netherlands), Pieter Jan van Doormaal (Erasmus MC University Medical Center, Rotterdam, the Netherlands), Geert Lycklama (Haaglanden Medical Center, the Hague, the Netherlands), Albert Yoo (Texas Stroke Institute, Plano, Texas, United States of America), Stefan Roosendaal (Erasmus MC University Medical Center, Rotterdam, the Netherlands), Anton Meijer (Radboud University Medical Center, Nijmegen, the Netherlands), Menno Krietemeijer (Catharina Hospital, Eindhoven, the Netherlands), Reinoud Bokkers (University Medical Center Groningen, the Netherlands), Anouk van der Hoorn (University Medical Center Groningen, the Netherlands), Dick Gerrits (Medisch Spectrum Twente, Enschede, the Netherlands), Adverse event committee: Robert van Oostenbrugge (Cardiovascular Research Institute Maastricht (CARIM), Maastricht University Medical Center, Maastricht, The Netherlands), Ben Jansen (Elisabeth-TweeSteden Hospital, Tilburg, the Netherlands), Bart van der Worp (University Medical Center Utrecht, Brain Center, Utrecht, the Netherlands), Outcome assessment committee: Sanne Manschot (Haaglanden Medical Center, the Hague, the Netherlands), Henk Kerkhof (Albert Schweitzer Hospital, Dordrecht, the Netherlands), Peter Koudstaal (Amsterdam UMC, University of Amsterdam, Amsterdam, the Netherlands), Data management group: Hester Lingsma (Erasmus MC University Medical Center, Rotterdam, the Netherlands), Vicky Chalos (Erasmus MC University Medical Center, Rotterdam, the Netherlands), Olvert Berkhemer (Amsterdam UMC, University of Amsterdam, Amsterdam, the Netherlands, Erasmus MC University Medical Center, Rotterdam, the Netherlands), Imaging data management: Adriaan Versteeg (Erasmus MC University Medical Center, Rotterdam, the Netherlands), Lennard Wolff (Erasmus MC University Medical Center, Rotterdam, the Netherlands), Jiahang Su (Erasmus MC University Medical Center, Rotterdam, the Netherlands), Manon Tolhuisen (Amsterdam UMC, University of Amsterdam, Amsterdam, the Netherlands), Henk van Voorst (Amsterdam UMC, University of Amsterdam, Amsterdam, the Netherlands), Biomaterials and translational group: Hugo ten Cate (Cardiovascular Research Institute Maastricht (CARIM), Maastricht University Medical Center, Maastricht, The Netherlands), Moniek de Maat (Erasmus MC University Medical Center, Rotterdam, the Netherlands), Samantha Donse-Donkel (Erasmus MC University Medical Center, Rotterdam, the Netherlands), Heleen van Beusekom (Erasmus MC University Medical Center, Rotterdam, the Netherlands), Aladdin Taha (Erasmus MC University Medical Center, Rotterdam, the Netherlands), Local collaborators: Vicky Chalos (Erasmus MC University Medical Center, Rotterdam, the Netherlands), Kilian Treurniet (Amsterdam UMC, University of Amsterdam, Amsterdam, the Netherlands), Sophie van den Berg (Amsterdam UMC, University of Amsterdam, Amsterdam, the Netherlands), Rob van de Graaf (Erasmus MC University Medical Center, Rotterdam, the Netherland), Robert-Jan Goldhoorn (Cardiovascular Research Institute Maastricht (CARIM), Maastricht University Medical Center, Maastricht, The Netherlands), Wouter Hinsenveld (Cardiovascular Research Institute Maastricht (CARIM), Maastricht University Medical Center, Maastricht, The Netherlands), Anne Pirson (Cardiovascular Research Institute Maastricht (CARIM), Maastricht University Medical Center, Maastricht, The Netherlands), Lotte Sondag (Radboud University Medical Center, Nijmegen, the Netherlands), Rik Reinink (University Medical Center Utrecht, Brain Center, Utrecht, the Netherlands), Josje Brouwer (Amsterdam UMC, University of Amsterdam, Amsterdam, the Netherlands), Matthijs van der Sluijs (Erasmus MC University Medical Center, Rotterdam, the Netherlands), Sabine Collette (University Medical Center Groningen, the Netherlands), Wouter van der Steen (Erasmus MC University Medical Center, Rotterdam, the Netherlands), Miou Koopman (Amsterdam UMC, University of Amsterdam, Amsterdam, the Netherlands), Research nurses: Rita Sprengers (Amsterdam UMC, University of Amsterdam, Amsterdam, the Netherlands), Martin Sterrenberg (Erasmus MC University Medical Center, Rotterdam, the Netherlands), Naziha El Ghannouti (Erasmus MC University Medical Center, Rotterdam, the Netherlands), Sabrina Verheesen (Cardiovascular Research Institute Maastricht (CARIM), Maastricht University Medical Center, Maastricht, The Netherlands), Wilma Pellikaan (St. Antonius Hospital, Nieuwegein, the Netherlands), Kitty Blauwendraat (St. Antonius Hospital, Nieuwegein, the Netherlands), Yvonne Drabbe (HagaZiekenhuis, the Hague, the Netherlands), Joke de Meris (Haaglanden Medical Center, the Hague, the Netherlands), Michelle Simons (Rijnstate Hospital, Arnhem, the Netherlands), Hester Bongenaar (Catharina Hospital, Eindhoven, the Netherlands), Anja van Loon (Amphia Hospital, Breda, the Netherlands), Eva Ponjee (Isala Klinieken, Zwolle, the Netherlands), Rieke Eilander (Isala Klinieken, Zwolle, the Netherlands), Jasmijn Lodico (Medisch Spectrum Twente, Enschede, the Netherlands), Hanneke Droste (Medisch Spectrum Twente, Enschede, the Netherlands), Suze Kooij (Albert Schweitzer Hospital, Dordrecht, the Netherlands), Marieke de Jong (University Medical Center Groningen, the Netherlands), Esther Santegoets (Elisabeth-TweeSteden Hospital, Tilburg, the Netherlands), Suze Roodenburg (Albert Schweitzer Hospital, Dordrecht, the Netherlands), Ayla van Ahee (Amsterdam UMC, University of Amsterdam, Amsterdam, the Netherlands, University Medical Center Utrecht, Brain Center, Utrecht, the Netherlands), Marinette Moynier (Centre Hospitalier Universitaire de Montpellier, Montpellier, France), Annemie Devroye (University Hospitals Leuven, Leuven, Belgium), Evelyn Marcelis (University Hospitals Leuven, Leuven, Belgium), Ingrid Iezzi (Centre Hospitalier Chrétien, Liège, Belgium), Annie David (Centre Hospitalier Chrétien, Liège, Belgium), Atika Talbi (Pitié-Salpêtrière hospital APHP-Sorbonne Université Paris France, Paris, France), Study monitors: Leontien Heiligers (Erasmus MC University Medical Center, Rotterdam, the Netherlands), Yvonne Martens (Erasmus MC University Medical Center, Rotterdam, the Netherlands).
